# Condition Assessment of Foundation Piles and Utility Poles Based on Guided Wave Propagation Using a Network of Tactile Transducers and Support Vector Machines

**DOI:** 10.3390/s17122938

**Published:** 2017-12-18

**Authors:** Ulrike Dackermann, Yang Yu, Ernst Niederleithinger, Jianchun Li, Herbert Wiggenhauser

**Affiliations:** 1 Centre for Infrastructure Engineering and Safety, School of Civil and Environmental Engineering, Faculty of Engineering, University of New South Wales (UNSW), Sydney, NSW 2052, Australia; u.dackermann@unsw.edu.au; 2 Centre for Built Infrastructure Research, School of Civil and Environmental Engineering, Faculty of Engineering and Information Technology, University of Technology, Sydney, NSW 2007, Australia; jianchun.li@uts.edu.au; 3 Division 8.2, German Federal Institute for Material Research and Testing (BAM), 12205 Berlin, Germany; ernst.niederleithinger@bam.de (E.N.); herbert.wiggenhauser@bam.de (H.W.)

**Keywords:** Structural Health Monitoring, non-destructive testing, sensor network, tactile transducers, guided waves, support vector machine, principal component analysis, foundation piles, utility poles, pipelines

## Abstract

This paper presents a novel non-destructive testing and health monitoring system using a network of tactile transducers and accelerometers for the condition assessment and damage classification of foundation piles and utility poles. While in traditional pile integrity testing an impact hammer with broadband frequency excitation is typically used, the proposed testing system utilizes an innovative excitation system based on a network of tactile transducers to induce controlled narrow-band frequency stress waves. Thereby, the simultaneous excitation of multiple stress wave types and modes is avoided (or at least reduced), and targeted wave forms can be generated. The new testing system enables the testing and monitoring of foundation piles and utility poles where the top is inaccessible, making the new testing system suitable, for example, for the condition assessment of pile structures with obstructed heads and of poles with live wires. For system validation, the new system was experimentally tested on nine timber and concrete poles that were inflicted with several types of damage. The tactile transducers were excited with continuous sine wave signals of 1 kHz frequency. Support vector machines were employed together with advanced signal processing algorithms to distinguish recorded stress wave signals from pole structures with different types of damage. The results show that using fast Fourier transform signals, combined with principal component analysis as the input feature vector for support vector machine (SVM) classifiers with different kernel functions, can achieve damage classification with accuracies of 92.5% ± 7.5%.

## 1. Introduction

Structural Health Monitoring (SHM) is a wide and multi-disciplinary field dealing with innovative methods for monitoring structural safety, integrity and performance without affecting the operation of the structure [1]. For civil engineering structures, non-destructive testing (NDT) combined with active SHM presents the future in infrastructure management including condition assessment, monitoring and maintenance. For foundation piles and utility poles, inspection routines are still primarily based on traditional methods, including visual inspection, sounding and resistance drilling. While visual inspection is undoubtedly one of the oldest assessment methods, it is limited to accessible areas and surface damage, and like sounding and resistance drilling, its reliability and accuracy is highly dependent on the experience of the operator [2]. As an alternative to these limited low-tech condition assessment methods, guided wave-based methods, such as pile integrity testing, are established testing methods for concrete piles and deep foundations that provide objective quantitative data, and are able to potentially detect internal damage and evaluate the health condition of non-accessible areas such as embedded sections of foundation piles and utility poles [3,4,5]. In guided wave testing, stress waves are generated in a structure, typically through a sudden pressure or deformation caused by an impact excitation, which subsequently propagate through the structure similar to the propagation of sound waves through air [6]. In pile and pole structures, the wave types generated through guided wave testing are longitudinal, bending and Rayleigh waves. While longitudinal and bending waves are body waves that propagate inside the structure along hemispherical wave fronts, Rayleigh waves travel away from the disturbance along the surface. Due to the dependency of the wave’s velocity to the modulus of elasticity, Poisson’s ratio, density and geometry of the structure, analyzing stress waves can give indications of a structure’s soundness condition [7,8].

While stress-wave-based techniques have promising potentials in damage detection and health monitoring of foundation piles and utility poles, some important issues associated with the uncertainty of wave propagation in in-service structures must be considered. These issues include the effect of soil embedment coupled with unknown soil and structure conditions below ground line; the generation of different wave modes due to broadband frequency excitation; the complexity of the structure’s material properties, which can be non-homogeneous (reinforced concrete) or anisotropic (timber), and can include inhomogeneities such as small voids, honey-comb or natural defects; the sensitivity to environmental changes such as moisture and temperature fluctuations; and the influence of the impact location [9]. As such, in pile integrity testing, the excitation is typically applied at the top of the pile, which will generate pure longitudinal waves. For the testing of in-situ poles, however, which are typically 10 m to 12 m long with a soil embedment of 1 m to 1.6 m, a top excitation is not feasible. Hence, a side impact must be performed, resulting in more complex wave propagation and response wave patterns. Here, longitudinal waves, bending waves and Rayleigh waves will be generated at the same time. Due to the broadband excitation from an impact hammer, various wave modes of the different wave types will be generated simultaneously. Understanding the wave propagation behavior in pile and pole structures is essential for the design of testing techniques and for the analysis of stress wave measurements for condition assessment and damage detection. Due to the vast complexity of the wave propagation, innovative testing and advanced signal processing techniques must be applied to deal with the issues described above. Using machine learning techniques for the condition assessment and monitoring of foundation piles and utility poles has previously provided promising results for in-field integrity testing [3,10] and is further explored in this study.

This paper proposes a novel testing and analysis system that uses a network of low-cost tactile transducer technology from the audio industry (instead of a broadband frequency excitation using an impact hammer) to induce controlled narrow-band frequency stress wave signals, avoiding or at least minimizing the generation of multiple wave types and modes. A network of accelerometers, arranged in three rings, is used to capture the wave propagation along the pile or pole structure. The innovative system is tested on a number of undamaged and damaged timber and concrete poles in the laboratory and in the field (with soil embedment), with controllable and axisymmetric excitation. For signal processing, fast Fourier transform (FFT) data combined with principal component analysis (PCA) are used to analyze the captured time-domain stress wave signals for features extraction. To evaluate the health condition and damage states of the poles, different types of classification models based on state-of-the art machine learning techniques using support vector machines (SVMs) are trained. The proposed method is based on the assumption that similar types of damage result in similar patterns in stress wave measurements, which can be extracted and identified using the method presented. The results of this study show that the proposed testing and analysis system has great potential to identify the damage condition of pile/pole structures.

## 2. New Wave Excitation System Based on Tactile Transducers

The proposed testing system utilizes low-cost tactile transducer technology from the audio industry for the controlled induction of narrow-band frequency stress waves in foundation piles and utility poles. A tactile transducer is an electro-mechanical device, very similar to a traditional audio speaker, which is mounted to a structure and driven by an amplifier. While traditional audio speakers transfer sound waves through the air, tactile transducers transfer vibrations/sound waves through the structure, making it possible to feel the sound. Thereby, tactile transducers are able to induce controlled stress waves into a structure (such as a pole). This audio technology is very cost effective and readily available from Hi-Fi retailers. The tactile transducers employed in this study are Vidsonix tactile transducers (model VX-GH92) that have a frequency range of 0.35 to 16 kHz and an impedance of 6.9 Ohm at 400 Hz and a maximum power of 70 Watt (Vidsonix^®^ 2015).

As mentioned above, the motivation for adopting tactile transducers as excitation source for the condition assessment and monitoring of foundation piles and utility poles is that hammer excitation (the typical excitation source for previous NDT research related to pile and pole structures) has the disadvantage that only a broadband frequency range can be excited. Since a broadband frequency excitation results in the generation of different types and modes of stress waves, very complex wave propagation occurs in the pole structure. The complexity of wave behavior is aggravated for reinforced concrete and timber poles due to the non-homogeneous material characteristics, which result in complex wave propagation and dispersion effects of the travelling waves. A further disadvantage of a hammer excitation is that it cannot be standardized, as each operator applies the hammer hit with a different force, as well as variations in the impact duration and angle, which results in waves of different amplitudes and frequency components. In addition, due to the setup of in-situ pole structures, a hammer impact can only be imparted from the side (not the top) of a pole, resulting in an asymmetric wave excitation. Using tactile transducers as a wave excitation source for the NDT of foundation piles and utility poles can overcome the major issues associated with a hammer impact. For instance, a controlled narrow-band frequency range can be excited, the wave excitation can be standardized (no operator associated variations) and a mainly symmetric guided wave can be generated by using a circumferential network of tactile transducers. The use of an excitation ring system for the generation of axisymmetric guided waves (with the suppression of nonaxisymmetric modes) has been studied extensively for pipeline inspections and is a fairly established testing technique in this field [11].

**Figure 1:**
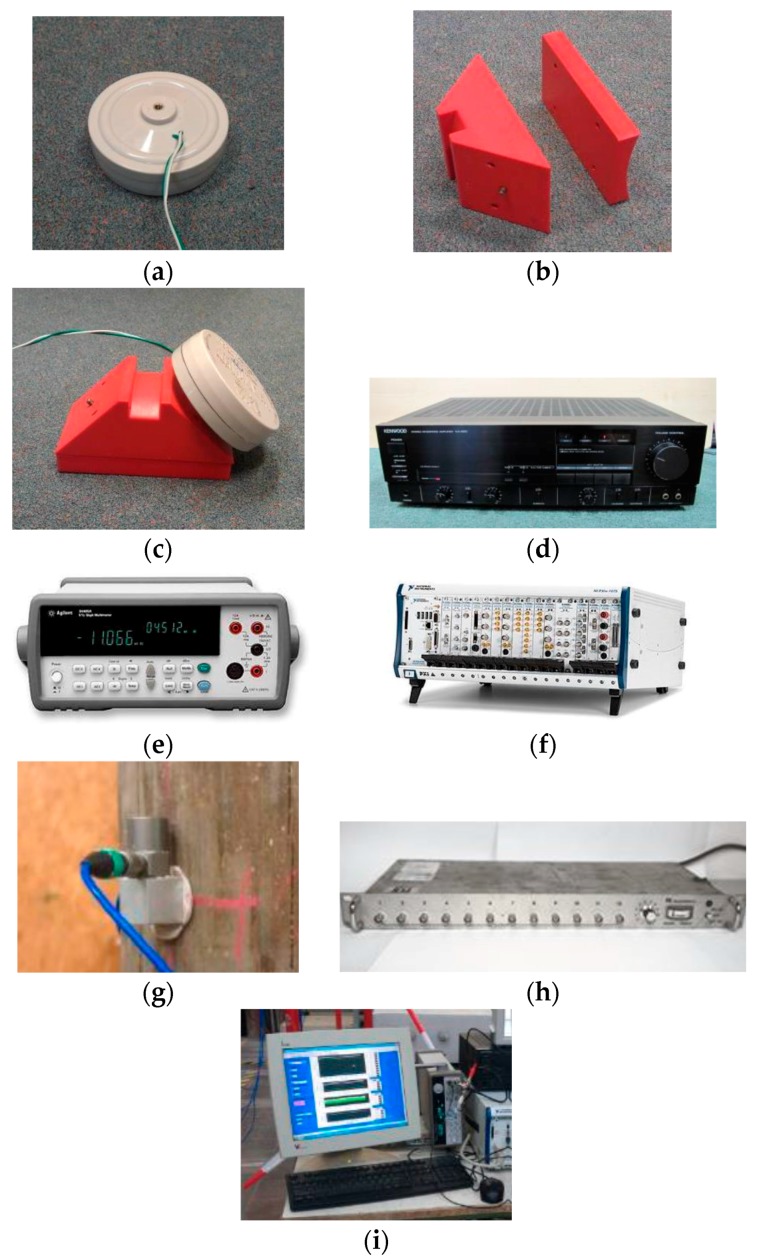
Testing equipment: (**a**) tactile transducer; (**b**) sensor wedge and curvature adapter; (**c**) tactile transducer mounted on sensor wedge; (**d**) Hi-Fi amplifier; (**e**) function generator; (**f**) data acquisition system; (**g**) accelerometer; (**h**) signal conditioner and (**i**) computer.

The testing equipment used for the experimental validation of the newly proposed testing system is presented in Figure 1 and includes the following: (a) a tactile transducer for a symmetric and synchronized narrow-band frequency excitation; (b) a sensor wedge (and curvature adapters) for directing the wave in the longitudinal direction of the structure; (c) a tactile transducer mounted on a sensor wedge; (d) a Hi-Fi amplifier for amplification and adjustment of the amplitude of the excited wave; (e) a function generator for generation of the desired excitation waveform and frequency; (f) a data acquisition system; (h) accelerometers (PCB, model 352C34) with a frequency range of 0.5 Hz to 10,000 Hz for measuring the wave propagation along the structure; (g) a signal conditioner for supplying the accelerometers with constant current; and (i) a computer for recording the data. 

## 3. Experimental Set-Up and Testing 

**Figure 2:**
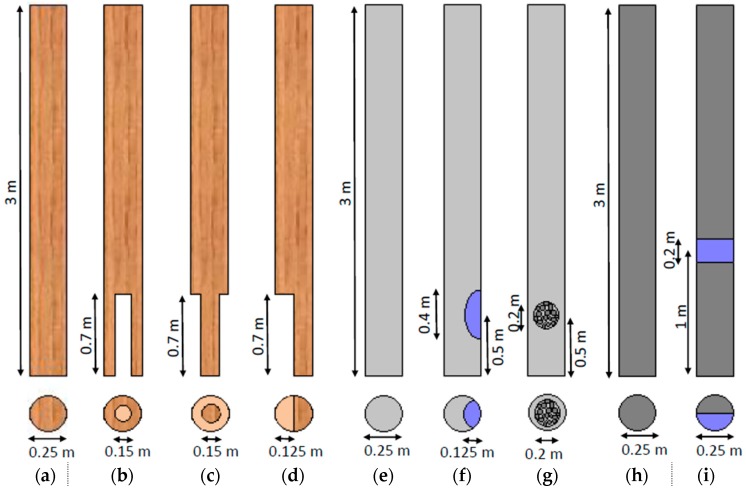
Dimensions and damage configurations of the tested poles in longitudinal and cross-sectional view: (**a**) undamaged timber pole, (**b**) timber pole with internal void damage, (**c**) timber pole with external circumferential cross-section loss damage, (**d**) timber poles with half-sided cross-section loss damage, (**e**) undamaged self-compacting concrete pole, (**f**) self-compacting concrete pole with surface void damage, (**g**) self-compacting concrete pole with internal honey-comb damage, (**h**) undamaged generic concrete pole, and (**i**) generic concrete pole with surface void damage.

**Figure 3:**
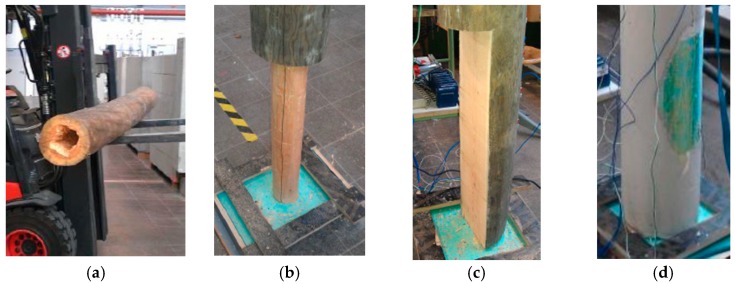
Examples of some damaged poles: (**a**) timber pole with internal void damage; (**b**) timber pole with external circumferential cross-section loss damage; (**c**) timber pole with half-sided cross-section loss damage and (**d**) self-compacting concrete pole with surface void damage.

To verify the new testing system and signal processing algorithm, laboratory and field testing was performed on nine poles. All poles tested had a length of 3 m and a diameter of 0.25 m. Four poles were made of timber (pine wood), three poles of self-compacting concrete (without steel reinforcement) and two poles of generic concrete (also without steel reinforcement). Of the timber poles, one pole was undamaged (TP1) and the other three poles were inflicted with three types of artificial damage simulating: (a) internal termite voids (TP2); (b) external circumferential fungi decay (TP3); and (c) half-sided fungi decay (TP4). Of the three self-compacting concrete poles, one was undamaged (SCP1), one had surface void damage and one internal honey-comb damage. Of the two generic concrete poles, one was undamaged and the other one had surface void damage. Figure 2 shows all tested poles presenting dimensions and damage types. Figure 3 displays photos of some example damage cases. The pole identifier, material, damage type and dimensions of all nine tested poles are listed in Table 1.

**Figure 4:**
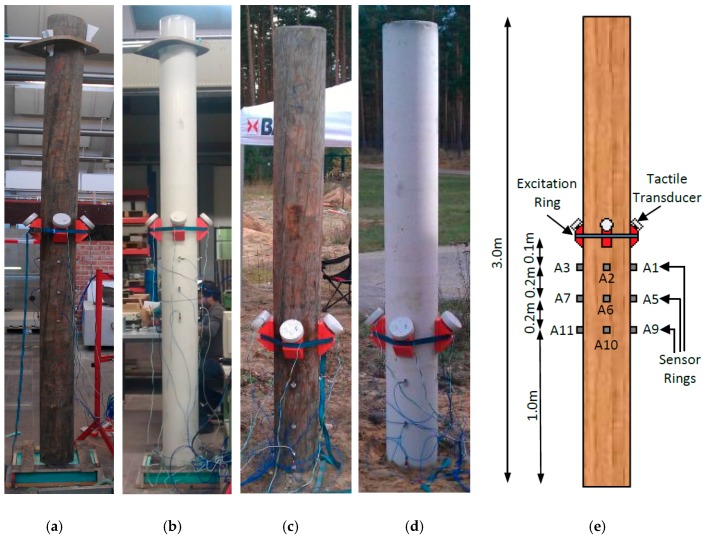
Laboratory and field testing set-up: (**a**) laboratory testing of timber pole; (**b**) laboratory testing of concrete pole; (**c**) field testing of timber pole; (**d**) field testing of concrete pole; and (**e**) labels and dimensions of test setup.

All nine poles were tested with two types of setup configurations, that is: (1) in the laboratory (standing freely on a Styrofoam mat); and (2) in the field (with soil embedment and exposure to environmental conditions). The laboratory testing was executed at the German Federal Institute for Material Research and Testing (BAM) non-destructive testing laboratories of Division 8.2, and the field testing at the BAM Test Site Technical Safety (BAM-TTS) in Horstwald. For all testing, four tactile transducers were used as excitation sources and 12 accelerometers (A1 to A12) measured the wave response of the structure. The four tactile transducers were mounted to the sensor wedges via a screw connection and were firmly attached to the pole structure at a height of 1.5 m in a ring formation with equal spacing using a ratchet strap. To allow full wave transmission from the sensor wedge to the pole, a thin layer of Vaseline was applied between the wedge and pole interface. The accelerometers were attached to the pole using moulding clay. For both laboratory and field testing setup, all accelerometers were attached in three rings with four accelerometers per ring. Sensor ring 1 was located 0.1 m below the excitation ring, and sensor rings 2 and 3 were distanced 0.3 m and 0.5 m below the excitation source, respectively. For field testing, the poles were embedded in the ground with a soil embedment of 1.0 m. As excitation force, continuous narrow-band sine wave signals with a frequency of 1 kHz were induced simultaneously at all four tactile transducers. The wave response of the pole structures was captured simultaneously by the 12 accelerometers with a sampling rate of 1 MHz and a recording time of 0.06 s. To generate multiple data sets, each pole configuration was tested 5 times. The detailed test setup arrangements for both experimental and field testing are shown in Figure 4.

## 4. Signal Processing: Feature Extraction Using FFT and PCA

**Figure 5:**
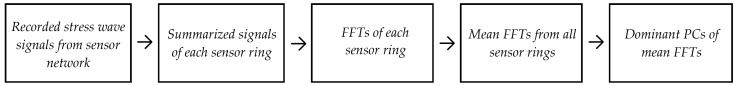
Flow-chart of feature extraction based on FFT signals and PCA.

For signal processing, an advanced signal analysis algorithm is proposed, which extracts damage features from the wave signals of the accelerometer network for the condition assessment of both timber and concrete pole structures using FFT data and PCA. In the proposed method, first, time-series stress wave signals are recorded from the laboratory and field testing of the different pole structures using the new testing system. For the testing, the poles are excited with narrow-band continuous wave signals in the shape of sine waves with an excitation frequency of 1 kHz. This frequency was chosen as it lies in the typical excitation range of traditional impact hammer testing. Second, the signals captured from accelerometers of the same ring are summed up to generate a new signal sequence. This signal summation is applied in order to suppress asymmetric wave components. Third, the newly generated time-domain signal sequence from each sensor ring is transferred to the frequency-domain using FFT and subsequently mean FFT signals are calculated by averaging the FFTs from the three sensor rings. Fourth, to reduce measurement noise effects and the amount of feature data, PCA is adopted to compress the mean FFT data. Finally, a selected number of the most dominant principal components (PCs) are chosen as damage specific indices distinguishing undamaged poles from different types of damaged poles. The detailed feature extraction procedure is presented in Figure 5.

**Figure 6:**
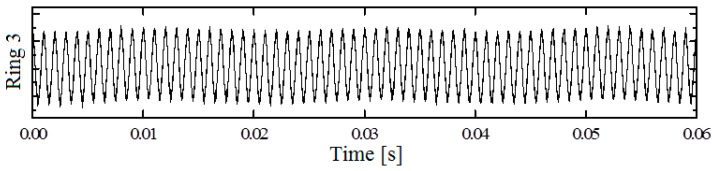
Original segmented time-domain acceleration signal from sensor ring 3 of a timber pole with internal void damage tested in the laboratory.

As an example of the summarized acceleration signals, Figure 6 presents a time-domain stress wave signal from sensor ring 3 of a timber pole with internal void damage tested in the laboratory. Signals from different sensor rings have similar wave patterns due to the continuous sine wave excitation (amplitude and frequency) with the exception of a phase shift resulting from the different ring positions with delayed up- and downward wave travel.

**Figure 7:**
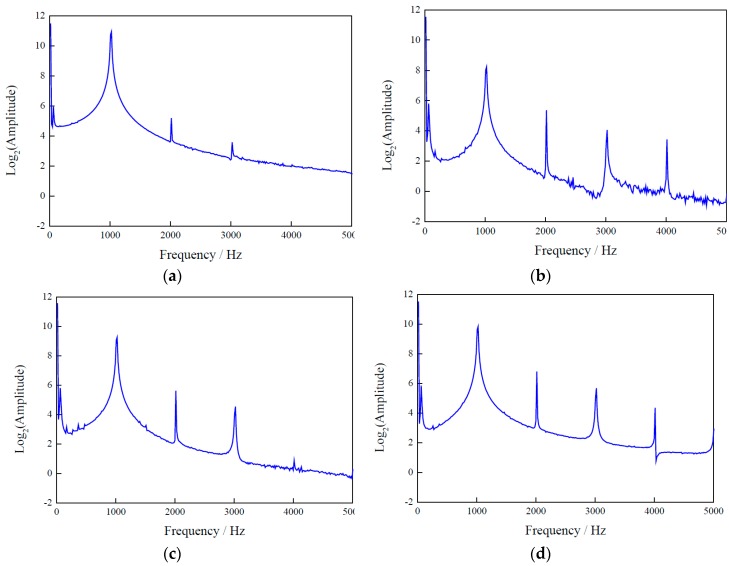
Averaged fast Fourier transforms (FFTs) of wave signals from the three sensor rings of timber poles tested in the laboratory: (**a**) TP1; (**b**) TP2; (**c**) TP3; and (**d**) TP4.

To extract damage-sensitive features from the measurement data, in the proposed method, the time-domain signals are transferred to the frequency domain using FFT. As an example, Figure 7 displays FFT data with a bandwidth of 0 to 5000 Hz of the four types of timber poles tested in the laboratory. It is noticeable that the energy distribution of the FFT signals from the intact pole is mainly concentrated around the 1 kHz frequency band (which is the excitation frequency of the transducers), while the response of the damaged poles contains higher amplitude harmonics at 2, 3 and 4 kHz. This suggests that the damage has introduced nonlinearities into the structure. In addition, different damage types produce different FFT amplitudes, in particular in the response at 1 kHz. These damage features can now be used by a classifier to identify the different types of damage.

If the full FFT data set is used as feature inputs for classifier training using machine learning techniques, it will result in slow training convergence and inefficient computations, since the full data set contains a vast amount of redundant information. Further, the FFT data contains disturbances from measurement noise, that are particularly present in field testing data, and which compromise the result accuracy. Due to these limitations, PCA is applied as an effective tool to filter noise and compress data by replacing the original FFTs with a small number of dominant PCs [12,13]. 

Pearson [14] originally developed the concept of PCA, which is based on the eigenvalue decomposition of the covariance matrix. PCA is a statistical method for achieving dimensionality reduction and is one of the most powerful multivariate data analysis techniques. Applying the concept of PCA, an original set of k variables is linearly transformed into a smaller set of n (n ≤ k) uncorrelated variables, the so-called principal components (PCs). The direction of the resulting eigenvectors represents the direction of the PCs and each PC is a linear combination of the original variables. After transformation, the PCs are weighted according to value of the corresponding eigenvalues. All the PCs are orthogonal to each other and form an orthogonal basis for the space of the data. So the first PC is weighted highest and has the largest eigenvalue. The corresponding first eigenvector represents the direction and amount of maximum variability of the original data set. The second PC is orthogonal to the first PC and represents the second most significant contribution in the data set, and so on [15]. By removing components that contribute least to the overall variance, the dimension of the original data set can drastically be reduced without significantly affecting the original data [16]. In addition to data reduction, PCA is also a powerful tool for filtering unwanted noise. As noise is a random feature that is not correlated with the global characteristics of the data set, it is represented by less significant PCs. Therefore, by ignoring PCs of low power, measurement noise is filtered.

**Figure 8:**
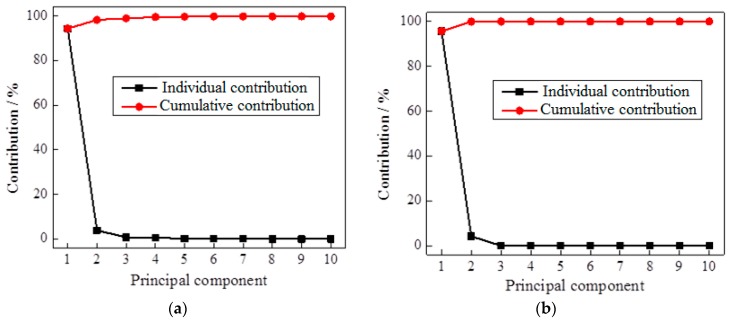
Contributions of the first ten principle components (PCs) of feature indices from timber pole specimens: (**a**) laboratory testing and (**b**) field testing.

For this study, PCA transformation is applied to the FFT signals of the tested pole structures. As example, Figure 8 displays the individual and cumulative contributions of the first ten PCs of the FFTs of timber poles tested in (a) the laboratory and (b) the field. It can be seen that the first four PCs contain more than 99% contribution of all the information. So by only selecting a small number of dominant PCs, the size of feature indices can be greatly reduced while maintaining the vast majority of information, which is highly beneficial for the classifier training. 

**Figure 9:**
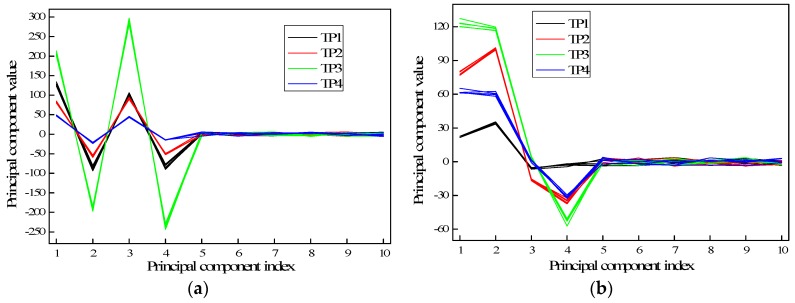
First ten PCs of FFT data of timber poles with different damage conditions: (**a**) laboratory testing and (**b**) field testing.

To select the optimal number of PCs, which contain sufficient information for damage classification, an investigation on the effect of the different damage types to the PCs is conducted. Figure 9 presents the first ten PCs of FFT data of the four different timber poles tested in (a) the laboratory and (b) the field. Since each pole configuration was tested five times, five lines are plotted for each pole. It can be observed that the first four components show clearly distinguishable patterns for the different types of damage, which forms the basis of the proposed damage identification method. Further, for damage of the same type, the PCs obtained are of similar values, that is, in this study, the PCs are similar for the five tests of the same pole. Since the PCs from the fifth component onwards have only small contribution values, which indicate their negligible contributions, the first four dominant components are considered as suitable indices for subsequent damage classification. It is noted that PCA was applied separately to the laboratory and the field testing data, since the boundary conditions are substantially different (soil embedment vs. free standing). Hence, the derived PCs are of different values reflecting the different composition of structural features. 

**Figure 10:**
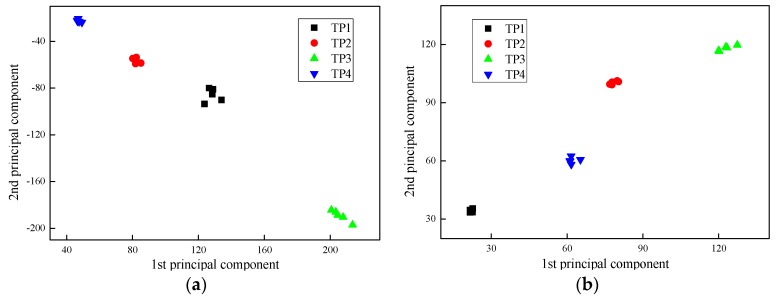
First ten PCs of FFT data of timber poles with different damage conditions: (**a**) laboratory testing and (**b**) field testing.

**Figure 11:**
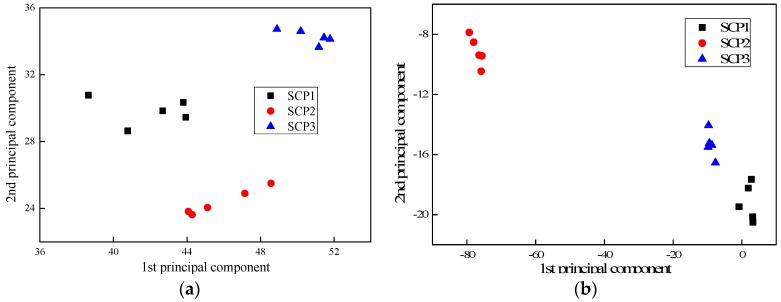
Principal component analysis (PCA) results of self-compacting concrete poles displaying the first two PCs: (**a**) laboratory testing and (**b**) field testing.

**Figure 12:**
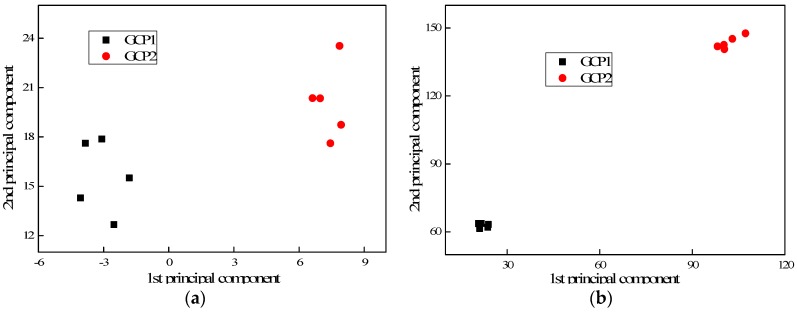
PCA results of generic concrete poles displaying the first two PCs: (**a**) laboratory testing and (**b**) field testing.

Figure 10, Figure 11 and Figure 12 present the PC results of the different pole structures showing only the first and second PCs. For each pole condition, all five test results are presented. From the figures, it can be seen that the PCs of the different pole types cluster together. This demonstrates that the selected indices are able to distinguish the different pole damage scenarios. It further shows that the proposed FFT and PCA-based signal processing method can effectively extract damage features from the wave signals.

## 5. Damage Classification Based on Support Vector Machine

After feature extraction from the raw stress wave signals, a classifier is set up for implementation of damage classification and condition assessment of the pole structures. In this study, the support vector machine (SVM) algorithm is selected to build the classifier due to its superior generalization ability and low sample requirement. The principle of SVM is that a set of training data is mapped from its original limited feature space to a much higher dimensional feature space using a mapping function [17,18]. Given a training data set with *l* elements $R=\{(x_{i},y_{i}),i=1,2,\ldots l\}$, each element in the set has a label to respond, denoted by y∈{−1,1}. If the training samples cannot be separated linearly in the feature space, the following expression is considered as objective function [19,20]:

(1) $$\begin{array}{l} \min\;\Phi (w)=\frac{1}{2}\langle w\cdot w\rangle +C\sum_{i=1}^{l}\xi_{i}\\ s.t.\;y_{i}(\langle w\cdot \Phi (x_{i})\rangle +b)\geq 1-\xi_{i},\;\xi_{i}\geq 0 \end{array}$$

where *C* denotes the penalty factor, *ξ_i_* (*i* = 1, 2, …, *l*) denote the slack parameters and *Φ* denotes the mapping function.

After adding a group of Lagrange multipliers, the optimization problem is described by:

(2) $$\begin{array}{l} \max\;L(\alpha )=\sum_{i=1}^{l}\alpha_{i}-\frac{1}{2}\sum_{i,j=1}^{l}\alpha_{i}\alpha_{j}y_{i}y_{j}k(x_{i},x_{j})\\ s.t.\;\sum_{i=1}^{l}\alpha_{i}y_{i}=0,\;0\leq \alpha_{i}\leq C \end{array}$$

where *α_i_* (*i* = 1, 2, …, *l*) denote the Lagrange multipliers. The decision function can be described as:

(3) $$f(x)=sign\left\{\sum_{i=1}^{l}y_{i}\alpha_{i}k(x_{i},x)+b\right\}$$

There are several commonly used kernel functions in SVM [21,22] including:

Linear function:                           $K\left(x_{i},x_{j}\right)=x_{i}^{T}x_{j}$
Polynomial function:                  $K\left(x_{i},x_{j}\right)={\left({\gamma x}_{i}^{T}x_{j}+r\right)}^{d},\;\gamma >0$
Radial basis function (RBF):      $K\left(x_{i},x_{j}\right)=\exp\left(-\gamma {\left||x_{i}-x_{j}|\right|}^{2}\right),\;\gamma >0$
Sigmoid function:                       $K\left(x_{i},x_{j}\right)=\tan h\left({\gamma x}_{i}^{T}x_{j}+r\right)$

In this work, all of the kernel functions above are used to train the SVM multi-classifier and the results of the different kernel functions are eventually compared against each other to select the optimal kernel function.

**Figure 13:**
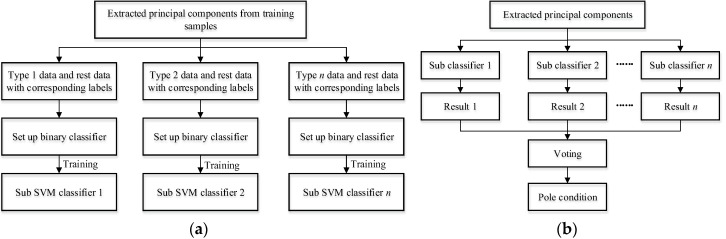
Set up and validation of multi-label classifier for damage condition assessment of pole structures: (**a**) classifier set up, and (**b**) voting strategy.

In general, SVM is used as a binary classifier to separate samples belonging to a negative or positive label. However, the damage identification of pole structures is a multi-label classification problem, and hence must be divided into several binary classification problems. In this work, the one-against-rest (OAR) method is adopted to set up multiple SVM classifiers with fewer calculation requirements [23]. For a number of *i* damage condition scenarios, the corresponding binary classifier is constructed to separate type *i* from the rest. Therefore, the number of the sub classifier depends on the number of condition categories to be identified from the tested poles. As discussed in the previous sections, the inputs of the classifier are the first four PCs from extracted FFT data of wave measurement signals while the output of the classifier is the pole damage condition label. Initially, the training samples are used to set up the multi-label classifier via machine learning. Then, the testing samples are employed to validate the performance of the trained classifier. Due to multiple sub classifiers resulting in multiple assessment results, a voting strategy is utilized to make a final decision; that is, the label with maximum votes gives the final result. If all results from the sub classifiers are labels of the rest, the pole condition is estimated as intact. The detailed training and validation procedure of the multi-label SVM classifier is depicted in Figure 13.

## 6. Results and Discussion

In this paper, training and validation of the proposed classifier is achieved by 5-fold cross-validation, in which all the extracted PCs from various damage condition scenarios are randomly divided into five groups with uniform data volume. The data of each group are left out in turns as the validation samples while the remaining samples are used as training samples to construct the classifier. The averaged classification accuracy, denoted by the ratio between correct predictions and total predictions, is used as indicator to verify the effectiveness of the trained classifier. In addition, the confusion matrix and Cohen’s Kappa value are employed to provide information on the distribution of the predicted label, which cannot be demonstrated using only the classification accuracy. The confusion matrix consists of rows and columns corresponding to the classification labels. Here, the columns denote the actual pole damage type and the rows denote the predicted pole damage type. Each component in the matrix indicates the number of correctly identified validation samples. The Cohen’s Kappa value is a statistical measure of the prediction distribution in the confusion matrix. The value varies from 0 to 1 with the maximum value representing the optimal prediction and with all values located at the diagonal line in the confusion matrix [24].

**Figure 14:**
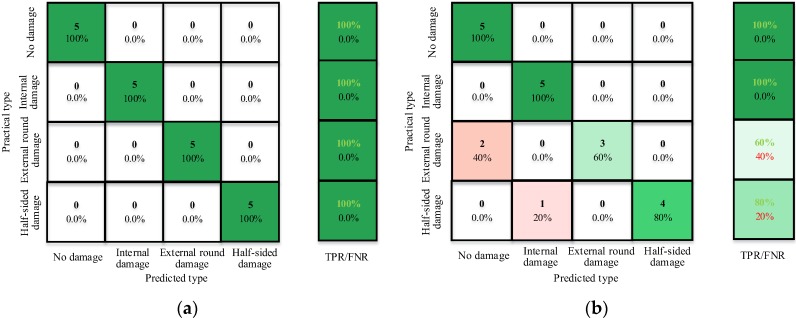
Confusion matrices of support vector machine (SVM) classifiers constructed from data of timber pole laboratory testing using: (**a**) the RBF kernel function; and (**b**) the linear kernel function.

As an example, Figure 14a,b present the results of the confusion matrix for the timber pole data from the laboratory testing using (a) the RBF function and (b) the linear kernel function, respectively. The results illustrate that the trained classifier can effectively separate the undamaged pole from the damaged poles with internal, external circumferential and half-sided damage with a classification accuracy of 100%. However, for the damage type identification, the classifier with the RBF kernel function outperforms the linear kernel function in the classification of the external circumferential and the half-sided damage. It can further be seen that the latter classifier predicts 40% of the poles with external circumferential damage as intact poles and 20% of the poles with half-sided damage as poles with internal damage.

**Figure 15:**
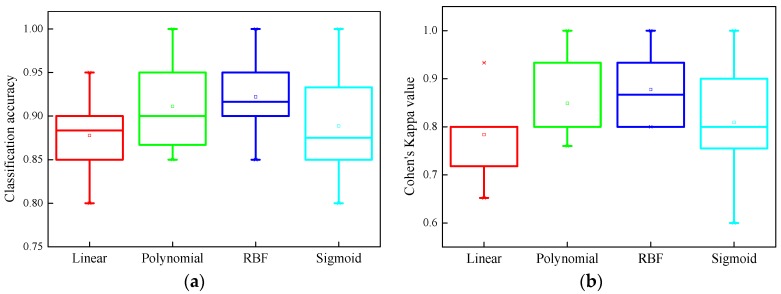
Statistical indicators for all tested pole specimen of the classifiers with different kernel functions: (**a**) classification accuracy; and (**b**) Cohen’s Kappa value.

Figure 15a shows the classification accuracies of all pole structures for different kernel function cases. The results demonstrate that the RBF function gives the best classification results compared to all other employed kernel functions. Using the RBF function can achieve classification accuracies of over 80%, meeting the practical requirement of pole structure condition assessment. Figure 15b displays the associated Cohen’s Kappa values of the classifiers with different kernel functions. Again, the RBF function outperforms the other kernel function. Overall, the results in Figure 15 show that the linear, polynomial and sigmoid functions can neither provide a statistically better classification accuracy nor a better Kappa value compared to the RBF function, which achieves classification accuracy results of 92.5% ± 7.5% and a Cohen’s Kappa value range of [0.8, 1.0]. 

Finally, the classification results for all pole specimens tested based on 5-fold cross validation are summarized in Table 2. It can be seen that the trained SVM-based multi-classifiers are able to identify the undamaged pole cases with 100% classification accuracy. Although several cases of damaged poles are inaccurately predicted, the models still achieve a very high performance (over 80%), which meets the condition assessment requirements of pole asset managements.

## 7. Conclusions

This paper presented a novel testing system for foundation piles and utility poles using a network of tactile transducers and accelerometers alongside an advanced signal processing technique for condition assessment and damage classification. The innovative testing system uses tactile transducers in a ring configuration to excite narrow-band frequency stress waves, in order to generate axisymmetric guided waves and thereby reduce the appearance of multi-wave types and modes typically encountered with broadband hammer excitation. The proposed advanced signal processing method utilized fast Fourier transform (FFT) signals and principal component analysis (PCA) to process measured stress wave signals containing specific damage signal features. The support vector machine (SVM) algorithm was used to set up multi-label classifiers for the prediction of pole damage conditions. To validate the new testing system and proposed signal analysis technique, different types of pole structures (timber and concrete poles) with various damage scenarios were tested in both the laboratory and the field (with soil embedment), inducing continuous sine wave excitation signals. The results of the advanced signal processing method demonstrated that the damage condition of the tested poles can be identified from extracted damage features using FFT data and PCA, and that the SVM multi-label classifier with the RBF kernel function can provide very good classification results with an optimal accuracy of 92.5% ± 7.5% and a Kappa value in the range of 0.8 and 1. In future work, the new narrow-band frequency excitation system will be tested in combination with the proposed advanced signal analysis technique for different excitation wave forms and frequencies, and it will be applied to actual in-situ foundation piles and utility poles with natural defects to verify the performance for practical engineering applications.

## Figures and Tables

**Table 1:** Pole identifier, material, damage type and dimensions of tested pole structures.

Identifier	Material	Damage Type	Damage Dimensions
TP1	Timber	Undamaged	-
TP2	Timber	Internal void damage	Φ = 0.15 m, h = 0.7 m
TP3	Timber	External circumferential damage	Φ = 0.15 m, h = 0.7 m
TP4	Timber	Half-sided cross-section loss damage	w = 0.125 m, h = 0.7 m
SCP1	Self-compacting concrete	Undamaged	-
SCP2	Self-compacting concrete	Surface void damage	wmax = 0.125 m, hmax = 0.4 m
SCP3	Self-compacting concrete	Internal honey-comb damage	Φ = 0.2 m
GCP1	Generic concrete	Undamaged	-
GCP2	Generic concrete	Surface void damage	w = 0.125 m, h = 0.2 m

**Table 2:** Summary of classification results for all pole specimens tested in both the laboratory and the field.

		Classification Accuracy (Correct/Total)			
**Type**		**Undamaged (TP1)**	**Internal Damage (TP2)**	**External Round (TP3)**	**Half Sided (TP4)**
**TP**	**Lab**	100% (5/5)	100% (5/5)	100% (5/5)	100% (5/5)
	**Field**	100% (5/5)	100% (5/5)	80% (4/5)	80% (4/5)
**Type**		**Undamaged (SCP1)**	**Surface void (SCP2)**		**Internal honey-comb (SCP3)**
**SCP**	**Lab**	100% (5/5)	80% (4/5)		100% (5/5)
	**Field**	100% (5/5)	80% (4/5)		80% (4/5)
**Type**		**Undamaged (GP1)**		**Surface void (GP2)**	
**GP**	**Lab**	100% (5/5)		100% (5/5)	
	**Field**	100% (5/5)		80% (4/5)	
